# Store-Operated Ca^2+^ Entry in Breast Cancer Cells: Remodeling and Functional Role

**DOI:** 10.3390/ijms19124053

**Published:** 2018-12-14

**Authors:** Isaac Jardin, Jose J. Lopez, Gines M. Salido, Juan A. Rosado

**Affiliations:** Department of Physiology, (Cellular Physiology Research Group), Institute of Molecular Pathology Biomarkers, University of Extremadura, 10003 Caceres, Spain; ijp@unex.es (I.J.); gsalido@unex.es (G.M.S.)

**Keywords:** STIM1, Orai1, TRPC channels, MCF7, MDA-MB-231, calcium entry, proliferation, migration, breast cancer

## Abstract

Breast cancer is the most common type of cancer in women. It is a heterogeneous disease that ranges from the less undifferentiated luminal A to the more aggressive basal or triple negative breast cancer molecular subtype. Ca^2+^ influx from the extracellular medium, but more specifically store-operated Ca^2+^ entry (SOCE), has been reported to play an important role in tumorigenesis and the maintenance of a variety of cancer hallmarks, including cell migration, proliferation, invasion or epithelial to mesenchymal transition. Breast cancer cells remodel the expression and functional role of the molecular components of SOCE. This review focuses on the functional role and remodeling of SOCE in breast cancer cells. The current studies suggest the need to deepen our understanding of SOCE in the biology of the different breast cancer subtypes in order to develop new and specific therapeutic strategies.

## 1. Molecular Basis of SOCE

Store-operated calcium entry (SOCE) is a major mechanism in non-excitable cells that, upon stimulation, finely modulates calcium (Ca^2+^) influx from the extracellular medium, leading to increases in cytosolic Ca^2+^ concentration ([Ca^2+^]_i_) required for the activation of a plethora of physiological functions, such as proliferation, exocytosis and gene transcription [1]. The main characters that modulate SOCE are the members of the STIM (stromal interaction molecule), Orai and TRPC (canonical transient receptor potential channel) protein families.

### 1.1. STIM, Orai and TRPC Proteins

STIM 1 is a 685-amino acid (aa) single-spamming membrane protein located both in internal vesicles, mainly the endoplasmic reticulum (ER), and the plasma membrane. The intraluminal region of STIM1 comprises a canonical and a hidden EF hand, which senses the ER Ca^2+^ concentration (K_d_ ~ 200–600 µM), and a sterile-α-motif (SAM), required by STIM1 dimerization [2]. Following the transmembrane (TM) domain, STIM1 cytosolic C-terminus contains several domains that will activate and regulate Orai (STIM1-Orai1 activation region, SOAR) and TRPC (STIM1 carboxyl terminus) proteins in the plasma membrane. The role of STIM1 as the Ca^2+^ sensor of the ER (and probably other agonist-sensitive Ca^2+^ stores [3]) and as the transient activator of the plasma membrane channels Orai and TRPC upon massive depletion of intracellular Ca^2+^ stores is well characterized (see [4,5,6] for more detailed review). In the same line, STIM2, more sensitive to low variations of intraluminal calcium levels, was proposed to mediate a lesser and prolonged SOCE activated to replenish marginally depleted Ca^2+^ stores [7]. However, the discovery of STIM2 variants, STIM2.1 (754 aa), STIM2.2 (746 aa) and STIM2.3 (599 aa), has introduced a new layer of complexity in the regulation of SOCE [8,9]. While STIM2.2 is responsible for the mechanism described above, STIM2.1 acts as an inhibitor of STIM1 and, subsequently, SOCE (see [8,9,10] for specific reviews).

The three members of the Orai family, Orai1 (301 aa), Orai2 (254 aa) and Orai3 (295 aa), are highly Ca^2+^-selective ion channels that mediate Ca^2+^ influx from the extracellular medium upon cell stimulation [11,12]. All of them express 4 TM domains, connected via one loop on the intracellular and two on the extracellular side with the N- and C-terminus located in the cytosol [13]. Both N- a C-terminus of Orai channel contain key domains for the association with and activation by STIM proteins [14,15,16]. Although Orai channels have been described to act in non-STIM1-activated mechanisms, such as the Kv10.1-Orai1 complex discussed in Section 3 [17], their main role is that of regulating Ca^2+^ influx upon intracellular Ca^2+^ store depletion and activation by STIM proteins. Thus, a homohexameric Orai1 [18], Orai2 or Orai3 channel, activated by STIM1 or STIM2, mediates both the highly selective Ca^2+^ released-activated Ca^2+^ (CRAC) channels with characteristic robust inwardly rectifying current [19], and, together with TRPC proteins, the less selective store-operated Ca^2+^ (SOC) channels [20,21,22,23]. It is still not clear how SOC channels operate; therefore, two models have been proposed: (a) both Orai and TRPC proteins form independent channels that are activated by STIM proteins [21,24], or (b) Orai and TRPC subunits form a heterochannel triggered by STIM1 or STIM2 [25]. Furthermore, 3 subunits of Orai1 and 2 subunits of Orai3 may form a store-independent pentameric channel activated by arachidonic acid (ARC) and regulated by the plasma membrane resident STIM1 and the store-operated Ca^2+^ entry-associated regulatory factor (SARAF) [26,27,28]. SARAF is an ER and plasma membrane resident STIM1 regulator that modulates resting [Ca^2+^]_i_, and participates in slow Ca^2+^ -dependent inactivation of SOCE, thus preventing Ca^2+^ overload [29,30,31,32,33]. Finally, the recent identification of two different forms of Orai1, Orai1α (301 aa) and Orai1β (237 aa), has opened new ways of understanding the complexity of SOCE. Due to alternative translation initiation [34], the two forms present distinct properties and capabilities to form different channels. Both Orai1α and Orai1β support CRAC and SOC channels, whereas only Orai1α is able to form ARC channels [35].

All the 28 members of the human TRP protein superfamily are non-selective cation channels permeable to both monovalent and divalent ions, such as Na^+^ and Ca^2+^, and all of them present a similar architecture: six TM domains, with the pore-forming region between the 5^th^ and 6^th^, connected by intracellular and extracellular loops, and with cytosolic N- and C-terminus domains. The channel is comprised by 4 subunits that may form a homo- or hetero-tetramer [36,37]. The N-terminus region contains a varying number of ankyrin-repeat domains which are essential for channel assembly and modulation [38]. The C-terminus encloses important domains for the interaction with regulators, such as IP_3_ receptor [39] and calmodulin [40,41], or allosteric activation [42]. Among all TRP proteins just those belonging to the canonical TRP (TRPC) family, TRPC1-7, have been described to be involved in the formation of SOC channels, which are activated by STIM1 and STIM2 carboxyl-terminus upon Ca^2+^ store depletion and triggering, subsequently, Ca^2+^ influx from the extracellular medium [21,23,35,43,44]. It is well established that the participation of different TRPC subunits in the formation of SOC channels is highly dependent on the cell type and the pattern of expression of those TRPC subunits within the cells [37,43].

### 1.2. Activation of SOCE in Healthy Cells

Under resting conditions, [Ca^2+^]_i_ is maintained low (in the range of 100–200 nM) by the cells. However, the concentration within the ER and other intracellular Ca^2+^ stores reaches values of mM (0.4–1 mM), which serve as a limited source of Ca^2+^. Furthermore, the concentration in the extracellular medium is even higher (1.8–2 mM) and provides an unlimited supply of Ca^2+^. Such huge differences in Ca^2+^ concentration establish an enormous gradient that should be finely regulated by the cells [45]. As described above, small variations in the intraluminal Ca^2+^ store concentration are regulated by STIM2.2. However, upon cell stimulation, there is an increase in [Ca^2+^]_i_ that triggers the physiological response. This increase begins with the depletion of intracellular Ca^2+^ stores, thus, the dissociation of Ca^2+^ from the EF hand domain of STIM1 [2]. Next, STIM1 undergoes a conformational change, starting with the SAM domains dimerization in the intraluminal N-terminal region [2]. The energy is then transferred through the TM domains to the cytosolic region of STIM1 [46], which unbends and exposes the Orai- and TRPC-activating regions of STIM1 [14,15,16]. Simultaneously, the recently described modulator of SOCE, EF-hand domain family member B (EFHB), displaces SARAF from STIM1, promoting the association of the latter with Orai1 and TRPC, and therefore the activation of the channels [47]. Finally, the influx of Ca^2+^ through CRAC and/or SOC channels will trigger the physiological responses. Later on, the stores are refilled and STIM1 is inactivated by Ca^2+^ re-association to its free EF-hand domain, which, in turn, triggers the dissociation from Orai1 and TRPC channels, and, subsequently, the bending of STIM1 to resting condition. Both processes are supported by SARAF, which displaces EFHB from STIM1 and maintains the latter in an idle position. Finally, STIM1 dissociation leads to channel closure at the plasma membrane, [Ca^2+^]_i_ returns to resting conditions and the cell becomes ready for further stimuli [1,13].

## 2. SOCE Remodeling in Breast Cancer

### 2.1. SOCE in Breast Cancer

In breast cancer cells, the regulation of [Ca^2+^]_i_ has been presented as crucial for tumorigenesis and the development of cancer hallmarks, including cell growth and proliferation, migration, metastasis and apoptosis resistance [48]. Consistent with this, the expression of a variety of Ca^2+^ channels is up-regulated in breast cancer cell lines and cancerous tissue, including Orai1, Orai3 and different TRP channels, such as TRPC6, TRPV6 and TRPM8, among others [49,50,51,52,53] (Table 1). In addition, intracellular Ca^2+^ mobilization and, more precisely, Ca^2+^ entry play important roles in angiogenesis. Consistent with this, in endothelial cells derived from human breast carcinomas, arachidonic acid (AA) has been reported to enhance Ca^2+^ influx and attenuate SOCE, which has been associated with the progression of the early phases of angiogenesis, including endothelial cell proliferation and tubulogenesis [54].

The role of Ca^2+^ influx in breast cancer cell biology has long been investigated. Initial in vitro studies by Yeh and coworkers in 1995 reported that the flavonoid quercetin and the Ca^2+^ channel blocker carboxyamidotriazole attenuate Ca^2+^ influx in the human MDA-MB-435 cell line, which, in turn, significantly impairs cell growth [55]. It is noteworthy to mention that despite MDA-MB-435 cells long having been used as a model for human breast cancer, several genetic studies, including gene expression analysis, CpG island promoter hypermethylation and miRNA expression, have revealed that the MDA-MB-435 cell line is a melanoma cell type [56]. More recently, it was reported that tranilast, an anti-allergic agent, attenuates Ca^2+^ influx and cytosolic Ca^2+^ oscillations evoked by insulin-like growth factor-1 (IGF-1) in the ER^+^ (estrogen receptor positive) breast cancer MCF7 cell line. Impairment of Ca^2+^ entry in MCF7 cells results in cell cycle arrest in the G1 phase [57], which supports the role of Ca^2+^ influx in cell proliferation. Calcium entry was also investigated in the human ER^-^ (estrogen receptor negative) BT-20 cell line by Sergeev and Rhoten [58], who reported the presence of a thapsigargin-sensitive intracellular Ca^2+^ store in these cells and a relevant receptor-operated and voltage-insensitive Ca^2+^ entry mechanism, which was found to be sensitive to La^3+^ and Ni^2+^ and permeable to Mn^2+^. Further studies performed in primary cultures of human normal and cancerous breast cells obtained from patients reported that angiotensin II is able to induce intracellular Ca^2+^ mobilization from TG-sensitive compartments, although store-operated or voltage-gated Ca^2+^ entry mechanisms were not detected [59].

Compelling evidence for the activation of SOCE in breast cancer cells was initially shown by Rossi and coworkers in the MCF7 cell line in response to ATP and TG [60]. In addition to Ca^2+^, SOCE in MCF7 cells was found to be permeable to Mn^2+^ and Sr^2+^. Interestingly, ATP-evoked Ca^2+^ influx, but not SOCE, was attenuated by cell treatment with 17β-estradiol [60]. The same group also reported functional activation of SOCE in the human HER2 overexpressing breast cancer SK-BR-3 cell line and the non-tumoral epithelial HBL100 cell line. In these cells, SOCE was mediated by two different pathways, sensitive and insensitive to low micromolar Gd^3+^ concentration [61].

**Figure 1:**
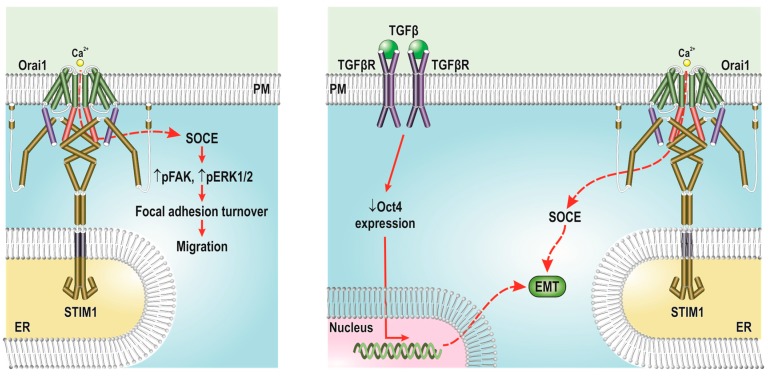
Functional role of SOCE in breast cancer cells. Left panel, in breast cancer cells, SOCE is involved in the phosphorylation status of the focal adhesion kinase (FAK) and ERK1/2, which is required for focal adhesion turnover and migration. Right panel, cell stimulation with TGFβ1, a potent inductor of epithelial to mesenchymal transition (EMT), leads to attenuated Oct4 expression, which, in turn, is associated with increased expression of STIM1 and Orai1 and enhanced SOCE. ER, endoplasmic reticulum; PM, plasma membrane; FAK, focal adhesion kinase; ERK, extracellular signal–regulated kinase; TGFβ, transforming growth factor-β; EMT, epithelial to mesenchymal transition.

There is a growing body of evidence linking SOCE with a variety of breast cancer cell hallmarks, including cell survival, proliferation, migration and invasion. Evidence for the relevant role of SOCE in breast cancer cell biology comes from studies exploring the involvement of protein glycosylation in breast cancer cell progression and metastasis. STIM1 and Orai1 are two N-glycosylated proteins. A recent study by Gueder and coworkers revealed that treatment of the breast cancer MCF7 and MDA-MB-231 cell lines with the pseudo-C-octyl glycoside 2-oxa-3-oxocastanospermine derivatives (CO-OCS), inhibitors of α-glycosidase, significantly decreased the expression of STIM1 at the protein level and attenuated SOCE. CO-OCS did not alter the expression of Orai1. Treatment of MCF7 and MDA-MB-231 cells with CO-OCS reduced the expression of β1-integrin, as well as the phosphorylation rates of the focal adhesion kinase (FAK) and ERK1/2, which results in the inhibition of cell migration without having any detectable effect in cell proliferation (Figure 1). In contrast, in the non-tumoral MCF10A cell line, treatment with CO-OCS was unable to alter the expression of STIM1 or Orai1, which indicates that protein glycosylation plays a relevant role in STIM1 expression and breast cancer cell migration [62].

SOCE has also been reported to play a role in breast cancer cell metabolism. In vitro studies performed by Tang and coworkers have reported that the mitochondrial Ca^2+^ uniporter (MCU) plays a critical role in SOCE and energy metabolism, which, in turn, are essential for breast cancer cell migration [63]. MCU silencing using siRNA or inhibition by ruthenium red has been reported to result in impairment of SOCE as well as migration in triple negative MDA-MB-231 breast cancer cells [63].

Soon after the identification of STIM1 and Orai1 as the key elements of SOCE, Yang et al. reported that these proteins are essential for in vitro MDA-MB-231 cell migration and invasion, as well as for in vivo MDA-MB-231 cell metastasis in immunodeficient NOD/SCID mice [64]. In vitro studies have reported that SOCE inhibition leads to MDA-MB-231 breast cancer cells with slower focal adhesion turnover rates, which indicates that Ca^2+^ influx through STIM1 and Orai1 is essential for focal adhesion assembly and disassembly in breast cancer cells [64,65]. Consistent with this, transforming growth factor (TGF)-β induces cell cycle arrest at the G0/G1 phase and impair cell proliferation in MDA-MB-231 and MCF7 breast cancer cells by a mechanism that involves decreased STIM1 expression and, subsequently, SOCE [66]. Interestingly, a recent in vitro study by Emeriau et al. revealed that those tyrosine kinase inhibitors that were able to decrease SOCE, such as lapatinib and CP-724714, exhibit greater anti-proliferative activity than those inhibitors that had no effect on SOCE [67]. Further in vitro studies have revealed that pharmacological inhibition of SOCE by Synta66 and YM58483 (also known as BTP2) in MDA-MB-468 cells significantly impairs migration and proliferation induced by a number of agonists, such as ATP, trypsin or EGF [68]. Conversely, both in vitro and in vivo studies have revealed that high Na^+^ concentration in the breast tumor microenvironment, which has been demonstrated by non-invasive magnetic resonance imaging [69] and induces inflammatory and cell proliferative responses [70], results in the activation of SOCE by a mechanism involving salt inducible kinase-3 up-regulation [71]. Enhanced SOCE results in overexpression of P-glycoprotein [71], which is one of the well-known mechanisms by which breast cancer cells develop resistance to chemotherapeutic drugs [72].

Further in vitro and in vivo studies supporting a relevant role for SOCE in breast cancer tumorigenesis have revealed that up-regulation of the angiotensin-converting enzyme-2/angiotensin-(1-7)/Mas axis, an important component of the tumor microenvironment, inhibits SOCE and the PAK1/NF-κB/Snail1 pathways and results in attenuated breast cancer cell migration and metastasis [73]. More recently, Chakraborty and coworkers have reported that phemindole, a synthetic di-indole derivative with anti-carcinogenic activity in triple negative breast cancer cells, reduces SOCE by down-regulation of STIM1 expression [74]. In vitro experiments have revealed that phemindole attenuates STIM1-Orai1 co-immunoprecipitation and co-localization in MDA-MB-231 cells, thus resulting in attenuated SOCE, ER stress and cell death. Phemindole-induced triple negative breast cancer cell death was reverted by restoration of STIM1 expression, which strongly supports a role for SOCE in triple negative cell survival [74].

Increased Ca^2+^ influx through SOCE has been associated with the activation of epithelial to mesenchymal transition (EMT). Davis and coworkers have reported that STIM1 and Orai1-mediated SOCE is involved in EMT of breast cancer cells, an essential step in cancer metastasis [75]. More recent studies by Zhang et al. have revealed that STIM1 and STIM2 differentially mediate TGFβ-induced Ca^2+^ entry and EMT in the breast cancer MCF7 and MDA-MB-231 cell lines. Both STIM1 and STIM2 mediate SOCE in response to TGFβ, but STIM2 is also involved in the activation of a receptor-operated, store-independent, Ca^2+^ influx mechanism upon stimulation with TGFβ [76]. The mechanism underlying EMT in breast cancer cells includes the expression of the stem cell-related transcription factor Oct4, which is highly expressed in the less invasive and metastatic MCF7 cancer cell line, while the triple negative MDA-MB-231 cell line exhibits undetectable expression. Cell treatment with TGFβ1, a potent EMT inductor, resulted in attenuated Oct4 expression, which is associated with up-regulated expression of STIM1 and Orai1 and, thus, enhanced SOCE [77] (Figure 1). More recent in vitro studies have also provided evidence for a role of TRPC1 and STIM1 in the activation of EMT by TGFβ in murine mammary epithelial NMuMG cells [78]. A role for TRPC1 in EMT has also been reported in airway remodeling mediated by house dust mites via up-regulation of the signal transducer and activator of transcription 3 (STAT3) expression [79].

Orai1-mediated SOCE has also been found to be essential for hypoxia-induced migration, invasion and angiogenesis in triple negative breast cancer cell lines. Hypoxia up-regulates Orai1 through the activation of Notch1 signaling, which, in turn, is responsible for the conduction of SOCE that activates calcineurin-nuclear factor of activated T-cell 4 (NFAT4) [80].

Recent studies have reported that Orai channels are involved in resistance to chemotherapeutic drugs in breast cancer cells. Through a combination of bioinformatic analysis and in vitro experiments, Hasna and coworkers have found a correlation between Orai3 overexpression and chemoresistance in several breast cancer data sets. The authors have reported that Ca^2+^ influx via Orai3, which is overexpressed in breast cancer tissue from patients, induces down-regulation of the p53 tumor suppressor protein via the pro-survival PI3K/Sgk-1/Sek-1 pathway [81].

### 2.2. SOCE Remodeling in Breast Cancer Cells

**Figure 2:**
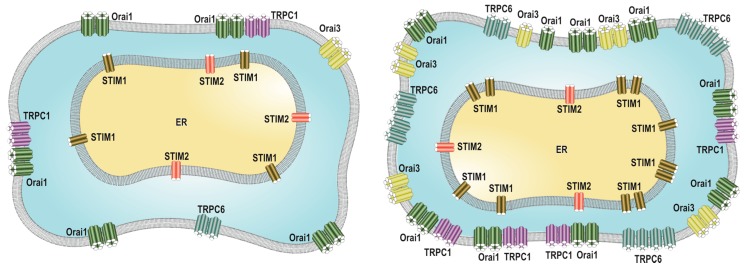
Overview of the remodeling of STIM, Orai and TRPC expression in breast cancer cells. Breast cancer cells (right panel) have been shown to overexpress Orai1 channels as compared to non-tumoral breast epithelial cells (left panel). Orai3 has been reported to be highly expressed in estrogen receptor positive (ER^+^) breast cancer cells. Concerning TRPC proteins, TRPC1 has been found to be modestly up-regulated in basal breast cancer cells, while TRPC6 is overexpressed both in ER^+^ and triple negative breast cancer cell lines. STIM1 mostly exhibits either high or normal expression in breast cancer cell lines as compared to non-tumoral cells; thus, the STIM1:STIM2 expression ratio has been found to be elevated in breast cancer cell lines and cancerous clinical samples. ER: endoplasmic reticulum.

Ca^2+^ channel remodeling is a feature of breast tissue, as illustrated by the enhanced Orai1 expression during lactation [49]. There is a growing body of evidence supporting the hypothesis that remodeling of the Ca^2+^ toolkit, schematized in Figure 2 and summarized in Table 1, represents a common signature of breast cancer cells underlying the development of breast cancer hallmarks. Changes in the expression of different Ca^2+^ channels might greatly alter the nature of the cellular responses to a variety of stimuli (reviewed in [65]), including growth factors. In the context of SOCE, Orai1 channels have mostly been reported to be overexpressed in a variety of breast cancer cell lines, including the widely studied cell lines ER^+^ MCF7 and the triple negative MDA-MB-231 breast cancer cells [49,82]. Concerning other Orai isoforms, different studies have demonstrated that the expression of Orai3 is elevated at the transcript and/or protein level in ER^+^ breast cancer cell lines and human clinical samples [50,82,83,87]. Analysis of the gene expression profile of TRPC1 in breast cancer-derived cell lines has reported that TRPC1 is modestly up-regulated in basal cell lines (including the representative MDA-MB-231 cells) compared with breast cancer cell lines of different subtypes or non-tumoral breast cell lines [86]. More recently, we have reported that TRPC6, which has also been found associated with SOCE [20,88,89], is overexpressed in MCF7 and MDA-MB-231 cells [82]. In these cells, TRPC6 plays a relevant role in the expression of Orai1 (in MDA-MB-231 cells) and Orai3 (in MCF7 cells) in the plasma membrane, an event that is required for the activation of SOCE and cell function. SOCE in MDA-MB-231 and MCF7 cells was drastically reduced after transfection with specific siRNA TRPC6 or expression plasmids for a pore-dead dominant-negative mutant of TRPC6 [82].

The expression level of STIM1 differs among the members of different breast cancer cell line subtypes. Among the triple negative cell lines, BT20 cells show STIM1 overexpression, while MDA-MB-231 or HCC 1937 cells appear to exhibit a normal expression level [83]. Similarly, ER^+^ breast cancer cell lines show either a normal (HCC 1500 or ZR751) or reduced (MCF7, T47D and BT474) expression of STIM1 [83]. Analysis of the relative expression of STIM1 and STIM2 revealed a STIM1:STIM2 expression ratio of between 2 and 5 in most breast cancer-derived cell lines investigated, except for the HER2 overexpressing cell line SK-BR-3, which exhibits an expression of STIM1 more than 10 times higher than that of STIM2 [49]. Similarly, breast cancerous clinical samples of the basal molecular subtype exhibit a high STIM1 and low STIM2 expression [49]. 

Remodeling of the expression profile of the different SOCE key elements in breast cancer cells has been demonstrated to play an important functional role. Motiani and coworkers confirmed that SOCE in triple negative MDA-MB-231 breast cancer cells is entirely dependent on STIM1 and Orai1, while in MCF7, SOCE is mediated by STIM1, STIM2 and Orai3 (overexpressed in this cell line), thus reporting important phenotypic differences between both cell lines [83]. Later on, the same group extended their observations, reporting that up-regulation of Orai3 in MCF7 cells depends on the expression of ERα and, consequently, Orai3 mediates ERα^+^ cell tumorigenesis in immunodeficient mice [84]. Down-regulation of Orai3 channels has been reported to arrest cell-cycle progression and to induce apoptosis in MCF7 cells but not in the non-tumoral MCF10A cell line cells with smaller Orai3 expression [50]. Orai3-dependent cell cycle progression has been associated with the activation of c-myc expression in tumor tissues and in the MCF7 cancer cell line through the MAP kinase pathway [90].

Specific remodeling of TRPC1 expression is also a feature of breast cancer cells. In vitro studies have revealed that TRPC1 expression is up-regulated during hypoxia-associated EMT in breast cancer cells. TRPC1 is required for the expression of the hypoxia-inducible factor 1α (HIF1α) in MDA-Mb-468 cells through the regulation of its translation and degradation rather than via transcriptional modulation. This mechanism involves an Akt phosphorylation-dependent pathway [86]. TRPC1 has also been reported to be involved in very specific aspects of EMT remodeling and induction [86]. The role of TRPC1 in the modulation of Akt phosphorylation has been confirmed by Kaemmerer et al. in the ER^-^/HER2^+^ epithelial breast cancer HCC1569 cell line [85]. Consistent with a role for TRPC1 in EMT induction, silencing TRPC1 expression has been reported to impair the activation of EMT stimulated by TGFβ in the murine mammary epithelial NMuMG cell line; meanwhile, TRPC1 overexpression increased TGFβ-induced EMT in these cells [78], which strongly supports a role for TRPC1 in the induction of EMT in breast cells.

We have recently reported that TRPC6 overexpression in MCF7 and MDA-MB-231 breast cancer cell lines is essential for full activation of SOCE, as well as cell proliferation, migration and invasion [82]. Furthermore, our results indicate that TRPC6 is required for MCF7 and MDA-MB-231 cell survival. In vitro studies have revealed that treatment of MCF7 and MDA-MB-231 cells with the olive oil derived phenolic compound oleocanthal induces an initial activation of TRPC6 channels, and thus, Ca^2+^ influx, followed by down-regulation of TPRC6 expression, which, in turn, drastically attenuates cell viability. By contrast, olecanthal has no effect in non-tumoral MCF10A cells, which is consistent with the low TRPC6 expression and dependency in these cells [53].

## 3. Overview of Other Orai and TRPC-Dependent Ca^2+^ Influx in Breast Cancer Cells

In addition to SOCE, other mechanisms for Ca^2+^ influx through Orai and TRPC channels have been reported to play a functional role in breast cancer cells. It is well known that membrane depolarization, as a result of Ca^2+^ influx itself, reduces the driving force and limit Ca^2+^ entry [91,92]. Ca^2+^-activated K^+^ channels (KCa) have been reported to contribute to sustain Ca^2+^ entry by inducing membrane repolarization/hyperpolarization upon Ca^2+^ entry [93]. For the fine regulation of Ca^2+^ signaling, KCa and Ca^2+^ channels have been reported to associate in complexes both in excitable and non-excitable cells [94]. Interestingly, current evidence has demonstrated that KCa–Ca^2+^ channel complexes contribute to the development of cancer hallmarks, including cell proliferation, cell migration and metastasis. Through a combination of in vitro and in vivo studies, Chantome and coworkers have revealed a functional interaction between the KCa channel SK3 and Orai1 in human breast and prostate cancer cells that is required for cell migration and bone metastasis. SK3 expression was detected in cancer cells but not in non-tumoral breast or prostate epithelial cells. In cancer cells, SK3 co-localizes with Orai1 in plasma membrane lipid rafts where the complex operates independently of STIM1 as a constitutive Ca^2+^ entry pathway [95]. In the breast cancer cell line MDA-MB-435s, the SK3-Orai1 channel complex has been reported to be regulated by cAMP, as activation of the cAMP-protein kinase A (PKA) pathway significantly attenuates both SK3 and SK3-Orai1 complex activity, which, in turn, results in a decrease in Ca^2+^ influx and cancer cell migration [96]. In contrast to the constitutive Orai1-mediated Ca^2+^ entry observed in these cells, a functional SK3-Orai1-TRPC1 channel complex has been reported to mediate SOCE and cell migration in colon cancer cells [97]. In the colon cancer cell HCT-116 line, activation of STIM1 by Ca^2+^ store depletion, using TG, results in the recruitment of Orai1 and TRPC1 into lipid raft domains containing SK3 channels. In this context, SOCE through Orai1 and TRPC1 channels has been found to be amplified by the SK3 channel activity [97].

A functional interaction between Orai1 channels and K_v_10.1 channels has also been reported to be involved in collagen-1-promoted breast cancer cell survival [17]. The breast cancer microenvironment is characterized by extensive collagen deposits, which promotes breast cancer initiation and progression [98]. A recent study has revealed that collagen 1 promotes in vitro breast cancer MCF7 cell survival through ERK1/2 phosphorylation and the overexpression and colocalization of K_v_10.1 and Orai1 ion channels, an interaction that enhances constitutive Ca^2+^ influx in these cells [17]. Similarly, a functional cooperation between TRPC1 and KCa3.1 has been reported to play a relevant role in basal Ca^2+^ entry in MCF7 cells suspended in the culture medium supplemented with 5% fetal bovine serum. The TRPC1-KCa3.1 interaction has been shown to be involved in MCF7 cell proliferation [99].

A store-independent mechanism for Ca^2+^ influx involving Orai1 and the secretory pathway Ca^2+^ ATPase-2 (SPCA2) plays an important role in Ca^2+^ uptake in mammary epithelial cells during lactation [100]. Both SPCA1 and SPCA2 are highly expressed in basal and luminal types breast tumors, respectively [100,101], where they might complex with Orai1 to elicit constitutive Ca^2+^ entry, which, in turn, might contribute to tumorigenesis [100,102].

## 4. Concluding Remarks

SOCE is an important mechanism for Ca^2+^ influx in breast cancer cells that supports several cancer hallmarks, including migration, proliferation and EMT. Breast cancer cells from the different subtypes undergo remodeling of the expression of specific molecular SOCE components, including Orai1, Orai3, TRPC1 and even TRPC6. Several SOCE inhibitors, including SKF-96365 and 2-APB, have been reported to attenuate in vitro several breast cancer hallmarks [66,78,103], in agreement with both in vivo and in vitro studies concerning other cancer types [104,105]. Impairment of SOCE by other synthetic compounds, such as phemindole [74], has proved that pharmacological tools addressed to SOCE inactivation suggest that SOCE constituents might be suitable therapeutic targets in breast cancer. A major challenge concerning Orai1 or STIM1 as potential therapeutic targets is the ubiquitous expression of both proteins and their crucial functional role; therefore, the pharmacological strategy should account with a cell-specific delivery mechanism. Alternatively, on the basis of specific SOCE features, such as the SOCE dependence on Orai3 in ER^+^ breast [83] or the up-regulation of TRPC6 and its specific role in Orai1/Orai3 plasma membrane expression in the MCF-7 and MDA-MB-231 cell lines [82], Orai3 or TRPC6 might be established as potential selective therapeutic targets for breast cancers. Analysis of the expression and functional role of these channels in breast cancer cells will undoubtedly provide valuable information about the biology of the different cancer subtypes and be the basis for the development of anti-cancer strategies. The development of a new generation of pharmacological tools against channels selectively expressed in different breast cancer subtypes, such as Orai3, might provide interesting results. 

## Figures and Tables

**Table 1:** SOCE constituents and remodeling in different breast cancer cell subtypes.

**Breast Cancer Subtype**	**Cell Line**	**SOCE Constituents**	**References**
Estrogen receptor positive	MCF7	STIM1, STIM2, Orai3 and TRPC6	[50,53,82,83,84]
Triple Negative/Basal	MDA-MB-231	STIM1, Orai1 and TRPC6	[53,82,83,84]
**Breast Cancer Subtype**	**Cell Line**	**SOCE Remodeling**	**References**
Estrogen receptor positive	MCF7	↓STIM1, ↔STIM2, ↑Orai3 and ↑TRPC6	[50,53,82,83,84]
	HCC 1500	↔STIM1	[83]
	ZR751	↔STIM1	[83]
	T47D	↓STIM1	[83]
	BT474	↓STIM1	[83]
HER2	HCC1569	↑TRPC1	[85]
Triple Negative/Basal	MDA-MB-231	↔STIM1, ↑Orai1, ↑TRPC1 and ↑TRPC6	[53,82,83,84,86]
	MDA-MB-468	↑TRPC1	[86]
	BT20	↑STIM1	[83]
	HCC 1937	↔STIM1	[83]
	Patients	↑STIM1, ↓STIM2 and ↑TRPC6	[49]

↔ Similar levels in cancer and non-tumoral cells. ↑ Increased levels in cancer cells. ↓ Decreased levels in cancer cells.

## References

[1] Putney J.W. (2011). The physiological function of store-operated calcium entry. Neurochem. Res..

[2] Stathopulos P.B., Zheng L., Li G.Y., Plevin M.J., Ikura M. (2008). Structural and mechanistic insights into STIM1-mediated initiation of store-operated calcium entry. Cell.

[3] Zbidi H., Jardin I., Woodard G.E., Lopez J.J., Berna-Erro A., Salido G.M., Rosado J.A. (2011). STIM1 and STIM2 Are Located in the Acidic Ca^2+^ Stores and Associates with Orai1 upon Depletion of the Acidic Stores in Human Platelets. J. Biol. Chem..

[4] Roos J., DiGregorio P.J., Yeromin A.V., Ohlsen K., Lioudyno M., Zhang S., Safrina O., Kozak J.A., Wagner S.L., Cahalan M.D. (2005). STIM1, an essential and conserved component of store-operated Ca^2+^ channel function. J. Cell. Biol..

[5] Zhang S.L., Yu Y., Roos J., Kozak J.A., Deerinck T.J., Ellisman M.H., Stauderman K.A., Cahalan M.D. (2005). STIM1 is a Ca^2+^ sensor that activates CRAC channels and migrates from the Ca^2+^ store to the plasma membrane. Nature.

[6] Fahrner M., Schindl R., Romanin C., Kozak J.A., Putney J.W. (2018). Studies of Structure-Function and Subunit Composition of Orai/STIM Channel. Calcium Entry Channels in Non-Excitable Cells.

[7] Brandman O., Liou J., Park W.S., Meyer T. (2007). STIM2 is a feedback regulator that stabilizes basal cytosolic and endoplasmic reticulum Ca^2+^ levels. Cell.

[8] Miederer A.M., Alansary D., Schwar G., Lee P.H., Jung M., Helms V., Niemeyer B.A. (2015). A STIM2 splice variant negatively regulates store-operated calcium entry. Nat. Commun..

[9] Rana A., Yen M., Sadaghiani A.M., Malmersjo S., Park C.Y., Dolmetsch R.E., Lewis R.S. (2015). Alternative splicing converts STIM2 from an activator to an inhibitor of store-operated calcium channels. J. Cell Biol..

[10] Berna-Erro A., Jardin I., Salido G.M., Rosado J.A. (2017). Role of STIM2 in cell function and physiopathology. J. Physiol..

[11] Feske S., Gwack Y., Prakriya M., Srikanth S., Puppel S.H., Tanasa B., Hogan P.G., Lewis R.S., Daly M., Rao A. (2006). A mutation in Orai1 causes immune deficiency by abrogating CRAC channel function. Nature.

[12] Prakriya M., Feske S., Gwack Y., Srikanth S., Rao A., Hogan P.G. (2006). Orai1 is an essential pore subunit of the CRAC channel. Nature.

[13] Derler I., Madl J., Schutz G., Romanin C. (2012). Structure, regulation and biophysics of I(CRAC), STIM/Orai1. Adv. Exp. Med. Biol..

[14] Muik M., Fahrner M., Derler I., Schindl R., Bergsmann J., Frischauf I., Groschner K., Romanin C. (2009). A Cytosolic Homomerization and a Modulatory Domain within STIM1 C Terminus Determine Coupling to ORAI1 Channels. J. Biol. Chem..

[15] Park C.Y., Hoover P.J., Mullins F.M., Bachhawat P., Covington E.D., Raunser S., Walz T., Garcia K.C., Dolmetsch R.E., Lewis R.S. (2009). STIM1 clusters and activates CRAC channels via direct binding of a cytosolic domain to Orai1. Cell.

[16] Yuan J.P., Zeng W., Dorwart M.R., Choi Y.J., Worley P.F., Muallem S. (2009). SOAR and the polybasic STIM1 domains gate and regulate Orai channels. Nat. Cell. Biol..

[17] Badaoui M., Mimsy-Julienne C., Saby C., Van Gulick L., Peretti M., Jeannesson P., Morjani H., Ouadid-Ahidouch H. (2018). Collagen type 1 promotes survival of human breast cancer cells by overexpressing Kv10.1 potassium and Orai1 calcium channels through DDR1-dependent pathway. Oncotarget.

[18] Hou X., Pedi L., Diver M.M., Long S.B. (2012). Crystal structure of the calcium release-activated calcium channel Orai. Science.

[19] Scrimgeour N., Litjens T., Ma L., Barritt G.J., Rychkov G.Y. (2009). Properties of Orai1 mediated store-operated current depend on the expression levels of STIM1 and Orai1 proteins. J. Physiol..

[20] Brechard S., Melchior C., Plancon S., Schenten V., Tschirhart E.J. (2008). Store-operated Ca^2+^ channels formed by TRPC1, TRPC6 and Orai1 and non-store-operated channels formed by TRPC3 are involved in the regulation of NADPH oxidase in HL-60 granulocytes. Cell. Calcium.

[21] Jardin I., Lopez J.J., Salido G.M., Rosado J.A. (2008). Orai1 mediates the interaction between STIM1 and hTRPC1 and regulates the mode of activation of hTRPC1-forming Ca^2+^ channels. J. Biol. Chem..

[22] Kim M.S., Zeng W., Yuan J.P., Shin D.M., Worley P.F., Muallem S. (2009). Native Store-operated Ca^2+^ Influx Requires the Channel Function of Orai1 and TRPC1. J. Biol. Chem..

[23] Ong H.L., Cheng K.T., Liu X., Bandyopadhyay B.C., Paria B.C., Soboloff J., Pani B., Gwack Y., Srikanth S., Singh B.B. (2007). Dynamic assembly of TRPC1-STIM1-Orai1 ternary complex is involved in store-operated calcium influx. Evidence for similarities in store-operated and calcium release-activated calcium channel components. J. Biol. Chem..

[24] Cheng K.T., Liu X., Ong H.L., Swaim W., Ambudkar I.S. (2011). Local Ca^2+^ entry via Orai1 regulates plasma membrane recruitment of TRPC1 and controls cytosolic Ca^2+^ signals required for specific cell functions. PLoS Biol..

[25] Ong E.C., Nesin V., Long C.L., Bai C.X., Guz J.L., Ivanov I.P., Abramowitz J., Birnbaumer L., Humphrey M.B., Tsiokas L. (2013). A TRPC1 protein-dependent pathway regulates osteoclast formation and function. J. Biol. Chem..

[26] Shuttleworth T.J., Thompson J.L., Mignen O. (2007). STIM1 and the noncapacitative ARC channels. Cell. Calcium.

[27] Shuttleworth T.J. (2009). Arachidonic acid, ARC channels, and Orai proteins. Cell. Calcium.

[28] Albarran L., Lopez J.J., Woodard G.E., Salido G.M., Rosado J.A. (2016). Store-operated Ca2+ entry-associated regulatory factor (SARAF) plays an important role in the regulation of arachidonate-regulated Ca^2+^ (ARC) channels. J. Biol. Chem..

[29] Albarran L., Lopez J.J., Gomez L.J., Salido G.M., Rosado J.A. (2016). SARAF modulates TRPC1, but not TRPC6, channel function in a STIM1-independent manner. Biochem. J..

[30] Palty R., Raveh A., Kaminsky I., Meller R., Reuveny E. (2012). SARAF inactivates the store operated calcium entry machinery to prevent excess calcium refilling. Cell.

[31] Albarran L., Regodon S., Salido G.M., Lopez J.J., Rosado J.A. (2016). Role of STIM1 in the surface expression of SARAF. Channels (Austin).

[32] Jha A., Ahuja M., Maleth J., Moreno C.M., Yuan J.P., Kim M.S., Muallem S. (2013). The STIM1 CTID domain determines access of SARAF to SOAR to regulate Orai1 channel function. J. Cell. Biol..

[33] Albarran L., Lopez J.J., Ben Amor N., Martín-Cano F.E., Berna-Erro A., Smani T., Salido G.M., Rosado J.A. (2016). Dynamic interaction of SARAF with STIM1 and Orai1 to modulate store-operated calcium entry. Sci. Rep..

[34] Fukushima M., Tomita T., Janoshazi A., Putney J.W. (2012). Alternative translation initiation gives rise to two isoforms of Orai1 with distinct plasma membrane mobilities. J. Cell. Sci..

[35] Desai P.N., Zhang X., Wu S., Janoshazi A., Bolimuntha S., Putney J.W., Trebak M. (2015). Multiple types of calcium channels arising from alternative translation initiation of the Orai1 message. Sci. Signal..

[36] Saotome K., Singh A.K., Yelshanskaya M.V., Sobolevsky A.I. (2016). Crystal structure of the epithelial calcium channel TRPV6. Nature.

[37] Pedersen S.F., Owsianik G., Nilius B. (2005). TRP channels: An overview. Cell. Calcium.

[38] Phelps C.B., Huang R.J., Lishko P.V., Wang R.R., Gaudet R. (2008). Structural analyses of the ankyrin repeat domain of TRPV6 and related TRPV ion channels. Biochemistry.

[39] Rosado J.A., Sage S.O. (2001). Activation of store-mediated calcium entry by secretion-like coupling between the inositol 1,4,5-trisphosphate receptor type II and human transient receptor potential (hTrp1) channels in human platelets. Biochem. J..

[40] Singh A.K., McGoldrick L.L., Twomey E.C., Sobolevsky A.I. (2018). Mechanism of calmodulin inactivation of the calcium-selective TRP channel TRPV6. Sci. Adv..

[41] Dionisio N., Albarran L., Berna-Erro A., Hernandez-Cruz J.M., Salido G.M., Rosado J.A. (2011). Functional role of the calmodulin- and inositol 1,4,5-trisphosphate receptor-binding (CIRB) site of TRPC6 in human platelet activation. Cell Signal..

[42] Gregorio-Teruel L., Valente P., Gonzalez-Ros J.M., Fernandez-Ballester G., Ferrer-Montiel A. (2014). Mutation of I696 and W697 in the TRP box of vanilloid receptor subtype I modulates allosteric channel activation. J. Gen. Physiol..

[43] Salido G.M., Jardin I., Rosado J.A. (2011). The TRPC Ion Channels: Association with Orai1 and STIM1 Proteins and Participation in Capacitative and Non-capacitative Calcium Entry. Adv. Exp. Med. Biol..

[44] Huang G.N., Zeng W., Kim J.Y., Yuan J.P., Han L., Muallem S., Worley P.F. (2006). STIM1 carboxyl-terminus activates native SOC, Icrac and TRPC1 channels. Nat. Cell. Biol..

[45] Berridge M.J., Bootman M.D., Roderick H.L. (2003). Calcium signalling: dynamics, homeostasis and remodelling. Nat. Rev. Mol. Cell. Biol..

[46] Ma G., Wei M., He L., Liu C., Wu B., Zhang S.L., Jing J., Liang X., Senes A., Tan P. (2015). Inside-out Ca^2+^ signalling prompted by STIM1 conformational switch. Nat. Commun..

[47] Albarran L., Lopez J.J., Jardin I., Sanchez-Collado J., Berna-Erro A., Smani T., Camello P.J., Salido G.M., Rosado J.A. (2018). EFHB is a novel cytosolic Ca^2+^ sensor that modulates STIM1-SARAF interaction. Cell Physiol. Biochem..

[48] Cross B.M., Breitwieser G.E., Reinhardt T.A., Rao R. (2014). Cellular calcium dynamics in lactation and breast cancer: From physiology to pathology. Am. J. Physiol. Cell Physiol..

[49] McAndrew D., Grice D.M., Peters A.A., Davis F.M., Stewart T., Rice M., Smart C.E., Brown M.A., Kenny P.A., Roberts-Thomson S.J. (2011). ORAI1-mediated calcium influx in lactation and in breast cancer. Mol. Cancer Ther..

[50] Faouzi M., Hague F., Potier M., Ahidouch A., Sevestre H., Ouadid-Ahidouch H. (2011). Down-regulation of Orai3 arrests cell-cycle progression and induces apoptosis in breast cancer cells but not in normal breast epithelial cells. J. Cell Physiol..

[51] Bolanz K.A., Hediger M.A., Landowski C.P. (2008). The role of TRPV6 in breast carcinogenesis. Mol. Cancer Ther..

[52] Chodon D., Guilbert A., Dhennin-Duthille I., Gautier M., Telliez M.S., Sevestre H., Ouadid-Ahidouch H. (2010). Estrogen regulation of TRPM8 expression in breast cancer cells. BMC Cancer.

[53] Diez-Bello R., Jardin I., Lopez J.J., El Haouari M., Ortega-Vidal J., Altarejos J., Salido G.M., Salido S., Rosado J.A. (2018). (-)Oleocanthal inhibits proliferation and migration by modulating Ca^2+^ entry through TRPC6 in breast cancer cells. Biochim. Biophys. Acta Mol. Cell. Res..

[54] Fiorio Pla A., Grange C., Antoniotti S., Tomatis C., Merlino A., Bussolati B., Munaron L. (2008). Arachidonic acid-induced Ca^2+^ entry is involved in early steps of tumor angiogenesis. Mol. Cancer Res..

[55] Yeh Y.A., Herenyiova M., Weber G. (1995). Quercetin: synergistic action with carboxyamidotriazole in human breast carcinoma cells. Life Sci..

[56] Prasad V.V., Gopalan R.O. (2015). Continued use of MDA-MB-435, a melanoma cell line, as a model for human breast cancer, even in year, 2014. NPJ Breast Cancer.

[57] Nie L., Oishi Y., Doi I., Shibata H., Kojima I. (1997). Inhibition of proliferation of MCF-7 breast cancer cells by a blocker of Ca^2+^-permeable channel. Cell Calcium.

[58] Sergeev I.N., Rhoten W.B. (1998). Regulation of intracellular calcium in human breast cancer cells. Endocrine.

[59] Greco S., Elia M.G., Muscella A., Storelli C., Marsigliante S. (2002). AT1 angiotensin II receptor mediates intracellular calcium mobilization in normal and cancerous breast cells in primary culture. Cell Calcium.

[60] Rossi A.M., Picotto G., de Boland A.R., Boland R.L. (2002). Evidence on the operation of ATP-induced capacitative calcium entry in breast cancer cells and its blockade by 17beta-estradiol. J. Cell Biochem..

[61] Baldi C., Vazquez G., Boland R. (2003). Capacitative calcium influx in human epithelial breast cancer and non-tumorigenic cells occurs through Ca^2+^ entry pathways with different permeabilities to divalent cations. J. Cell Biochem..

[62] Gueder N., Allan G., Telliez M.S., Hague F., Fernandez J.M., Sanchez-Fernandez E.M., Ortiz-Mellet C., Ahidouch A., Ouadid-Ahidouch H. (2017). sp(2) -Iminosugar alpha-glucosidase inhibitor 1-C-octyl-2-oxa-3-oxocastanospermine specifically affected breast cancer cell migration through Stim1, beta1-integrin, and FAK signaling pathways. J. Cell Physiol..

[63] Tang S., Wang X., Shen Q., Yang X., Yu C., Cai C., Cai G., Meng X., Zou F. (2015). Mitochondrial Ca^2+^ uniporter is critical for store-operated Ca^2+^ entry-dependent breast cancer cell migration. Biochem. Biophys. Res. Commun..

[64] Yang S., Zhang J.J., Huang X.Y. (2009). Orai1 and STIM1 are critical for breast tumor cell migration and metastasis. Cancer Cell.

[65] Mo P., Yang S. (2018). The store-operated calcium channels in cancer metastasis: From cell migration, invasion to metastatic colonization. Front. Biosci..

[66] Cheng H., Wang S., Feng R. (2016). STIM1 plays an important role in TGF-beta-induced suppression of breast cancer cell proliferation. Oncotarget.

[67] Emeriau N., de Clippele M., Gailly P., Tajeddine N. (2018). Store operated calcium entry is altered by the inhibition of receptors tyrosine kinase. Oncotarget.

[68] Azimi I., Bong A.H., Poo G.X.H., Armitage K., Lok D., Roberts-Thomson S.J., Monteith G.R. (2018). Pharmacological inhibition of store-operated calcium entry in MDA-MB-468 basal A breast cancer cells: Consequences on calcium signalling, cell migration and proliferation. Cell Mol. Life Sci..

[69] Ouwerkerk R., Jacobs M.A., Macura K.J., Wolff A.C., Stearns V., Mezban S.D., Khouri N.F., Bluemke D.A., Bottomley P.A. (2007). Elevated tissue sodium concentration in malignant breast lesions detected with non-invasive 23Na MRI. Breast Cancer Res. Treat..

[70] Amara S., Ivy M.T., Myles E.L., Tiriveedhi V. (2016). Sodium channel gammaENaC mediates IL-17 synergized high salt induced inflammatory stress in breast cancer cells. Cell. Immunol..

[71] Babaer D., Amara S., Ivy M., Zhao Y., Lammers P.E., Titze J.M., Tiriveedhi V. (2018). High salt induces P-glycoprotein mediated treatment resistance in breast cancer cells through store operated calcium influx. Oncotarget.

[72] Callaghan R., Luk F., Bebawy M. (2014). Inhibition of the multidrug resistance P-glycoprotein: Time for a change of strategy?. Drug Metab. Dispos..

[73] Yu C., Tang W., Wang Y., Shen Q., Wang B., Cai C., Meng X., Zou F. (2016). Downregulation of ACE2/Ang-(1-7)/Mas axis promotes breast cancer metastasis by enhancing store-operated calcium entry. Cancer Lett..

[74] Chakraborty S., Ghosh S., Banerjee B., Santra A., Adhikary A., Misra A.K., Sen P.C. (2016). Phemindole, a Synthetic Di-indole Derivative Maneuvers the Store Operated Calcium Entry (SOCE) to Induce Potent Anti-Carcinogenic Activity in Human Triple Negative Breast Cancer Cells. Front. Pharmacol..

[75] Davis F.M., Peters A.A., Grice D.M., Cabot P.J., Parat M.O., Roberts-Thomson S.J., Monteith G.R. (2012). Non-stimulated, agonist-stimulated and store-operated Ca^2+^ influx in MDA-MB-468 breast cancer cells and the effect of EGF-induced EMT on calcium entry. PLoS ONE.

[76] Zhang S., Miao Y., Zheng X., Gong Y., Zhang J., Zou F., Cai C. (2017). STIM1 and STIM2 differently regulate endogenous Ca^2+^ entry and promote TGF-beta-induced EMT in breast cancer cells. Biochem. Biophys. Res. Commun..

[77] Hu J., Qin K., Zhang Y., Gong J., Li N., Lv D., Xiang R., Tan X. (2011). Downregulation of transcription factor Oct4 induces an epithelial-to-mesenchymal transition via enhancement of Ca^2+^ influx in breast cancer cells. Biochem. Biophys. Res. Commun..

[78] Schaar A., Sukumaran P., Sun Y., Dhasarathy A., Singh B.B. (2016). TRPC1-STIM1 activation modulates transforming growth factor beta-induced epithelial-to-mesenchymal transition. Oncotarget.

[79] Pu Q., Zhao Y., Sun Y., Huang T., Lin P., Zhou C., Qin S., Singh B.B., Wu M. (2018). TRPC1 intensifies house dust mite-induced airway remodeling by facilitating epithelial-to-mesenchymal transition and STAT3/NF-kappaB signaling. FASEB J..

[80] Liu X., Wang T., Wang Y., Chen Z., Hua D., Yao X., Ma X., Zhang P. (2018). Orai1 is critical for Notch-driven aggressiveness under hypoxic conditions in triple-negative breast cancers. Biochim. Biophys. Acta Mol. Basis Dis..

[81] Hasna J., Hague F., Rodat-Despoix L., Geerts D., Leroy C., Tulasne D., Ouadid-Ahidouch H., Kischel P. (2018). Orai3 calcium channel and resistance to chemotherapy in breast cancer cells: The p53 connection. Cell Death Differ..

[82] Jardin I., Diez-Bello R., Lopez J.J., Redondo P.C., Salido G.M., Smani T., Rosado J.A. (2018). TRPC6 Channels Are Required for Proliferation, Migration and Invasion of Breast Cancer Cell Lines by Modulation of Orai1 and Orai3 Surface Exposure. Cancers (Basel).

[83] Motiani R.K., Abdullaev I.F., Trebak M. (2010). A novel native store-operated calcium channel encoded by Orai3: Selective requirement of Orai3 versus Orai1 in estrogen receptor-positive versus estrogen receptor-negative breast cancer cells. J. Biol. Chem..

[84] Motiani R.K., Zhang X., Harmon K.E., Keller R.S., Matrougui K., Bennett J.A., Trebak M. (2013). Orai3 is an estrogen receptor alpha-regulated Ca^2+^ channel that promotes tumorigenesis. FASEB J..

[85] Kaemmerer E., Turner D., Peters A.A., Roberts-Thomson S.J., Monteith G.R. (2018). An automated epifluorescence microscopy imaging assay for the identification of phospho-AKT level modulators in breast cancer cells. J. Pharmacol. Toxicol. Methods.

[86] Azimi I., Milevskiy M.J.G., Kaemmerer E., Turner D., Yapa K., Brown M.A., Thompson E.W., Roberts-Thomson S.J., Monteith G.R. (2017). TRPC1 is a differential regulator of hypoxia-mediated events and Akt signalling in PTEN-deficient breast cancer cells. J. Cell Sci..

[87] Chalmers S.B., Monteith G.R. (2018). ORAI channels and cancer. Cell Calcium..

[88] Jardin I., Redondo P.C., Salido G.M., Rosado J.A. (2008). Phosphatidylinositol 4,5-bisphosphate enhances store-operated calcium entry through hTRPC6 channel in human platelets. Biochim. Biophys. Acta.

[89] Jardin I., Gomez L.J., Salido G.M., Rosado J.A. (2009). Dynamic interaction of hTRPC6 with the Orai1/STIM1 complex or hTRPC3 mediates its role in capacitative or non-capacitative Ca^2+^ entry pathways. Biochem. J..

[90] Faouzi M., Kischel P., Hague F., Ahidouch A., Benzerdjeb N., Sevestre H., Penner R., Ouadid-Ahidouch H. (2013). ORAI3 silencing alters cell proliferation and cell cycle progression via c-myc pathway in breast cancer cells. Biochim. Biophys. Acta.

[91] Jardin I., Albarran L., Salido G.M., Lopez J.J., Sage S.O., Rosado J.A. (2018). Fine-tuning of store-operated calcium entry by fast and slow Ca^2+^-dependent inactivation: Involvement of SARAF. Biochim. Biophys. Acta Mol. Cell Res..

[92] Launay P., Fleig A., Perraud A.L., Scharenberg A.M., Penner R., Kinet J.P. (2002). TRPM4 is a Ca^2+^-activated nonselective cation channel mediating cell membrane depolarization. Cell.

[93] Funabashi K., Ohya S., Yamamura H., Hatano N., Muraki K., Giles W., Imaizumi Y. (2010). Accelerated Ca^2+^ entry by membrane hyperpolarization due to Ca2+-activated K+ channel activation in response to histamine in chondrocytes. Am. J. Physiol. Cell Physiol..

[94] Gueguinou M., Chantome A., Fromont G., Bougnoux P., Vandier C., Potier-Cartereau M. (2014). KCa and Ca^2+^ channels: the complex thought. Biochim. Biophys. Acta.

[95] Chantome A., Potier-Cartereau M., Clarysse L., Fromont G., Marionneau-Lambot S., Gueguinou M., Pages J.C., Collin C., Oullier T., Girault A. (2013). Pivotal role of the lipid Raft SK3-Orai1 complex in human cancer cell migration and bone metastases. Cancer Res..

[96] Clarysse L., Gueguinou M., Potier-Cartereau M., Vandecasteele G., Bougnoux P., Chevalier S., Chantome A., Vandier C. (2014). cAMP-PKA inhibition of SK3 channel reduced both Ca^2+^ entry and cancer cell migration by regulation of SK3-Orai1 complex. Pflugers Arch..

[97] Gueguinou M., Harnois T., Crottes D., Uguen A., Deliot N., Gambade A., Chantome A., Haelters J.P., Jaffres P.A., Jourdan M.L. (2016). SK3/TRPC1/Orai1 complex regulates SOCE-dependent colon cancer cell migration: A novel opportunity to modulate anti-EGFR mAb action by the alkyl-lipid Ohmline. Oncotarget.

[98] Provenzano P.P., Inman D.R., Eliceiri K.W., Knittel J.G., Yan L., Rueden C.T., White J.G., Keely P.J. (2008). Collagen density promotes mammary tumor initiation and progression. BMC Med..

[99] Faouzi M., Hague F., Geerts D., Ay A.S., Potier-Cartereau M., Ahidouch A., Ouadid-Ahidouch H. (2016). Functional cooperation between KCa3.1 and TRPC1 channels in human breast cancer: Role in cell proliferation and patient prognosis. Oncotarget.

[100] Feng M., Grice D.M., Faddy H.M., Nguyen N., Leitch S., Wang Y., Muend S., Kenny P.A., Sukumar S., Roberts-Thomson S.J. (2010). Store-independent activation of Orai1 by SPCA2 in mammary tumors. Cell.

[101] Dang D., Prasad H., Rao R. (2017). Secretory pathway Ca^2+^-ATPases promote in vitro microcalcifications in breast cancer cells. Mol. Carcinog.

[102] Mignen O., Constantin B., Potier-Cartereau M., Penna A., Gautier M., Gueguinou M., Renaudineau Y., Shoji K.F., Felix R., Bayet E. (2017). Constitutive calcium entry and cancer: updated views and insights. Eur. Biophys. J..

[103] Guilbert A., Gautier M., Dhennin-Duthille I., Haren N., Sevestre H., Ouadid-Ahidouch H. (2009). Evidence that TRPM7 is required for breast cancer cell proliferation. Am. J. Physiol. Cell Physiol..

[104] Sun S., Li W., Zhang H., Zha L., Xue Y., Wu X., Zou F. (2012). Requirement for store-operated calcium entry in sodium butyrate-induced apoptosis in human colon cancer cells. Biosci Rep..

[105] Cai R., Ding X., Zhou K., Shi Y., Ge R., Ren G., Jin Y., Wang Y. (2009). Blockade of TRPC6 channels induced G2/M phase arrest and suppressed growth in human gastric cancer cells. Int. J. Cancer.

